# Assessment of prescribing competencies among final year medical students in Gezira State, Sudan

**DOI:** 10.1186/s12909-026-09129-3

**Published:** 2026-04-01

**Authors:** Sawsan Abdallateef, Bashir A. Yousef, Mohamed Elmustafa

**Affiliations:** 1 Department of Pharmacology, Faculty of Pharmacy, University of Gezira, Hospital Street, Wad Medani, Gezira State 21112 Sudan; 2 Department of Pharmacology, Faculty of Pharmacy, University of Khartoum, Khartoum, Khartoum State Sudan; 3 Department of Clinical Pharmacy and Pharmacology, Ibn Sina College for Medical Studies, Jeddah, Saudi Arabia; 4 Faculty of Pharmacy, Wad Medani University, Wad Medani, Gezira State Sudan

**Keywords:** Prescribing, Competence, Medical students, Clinical pharmacology, Therapeutics, Sudan

## Abstract

**Introduction:**

Prescribing medications is a critical part of medical practice, yet evidence indicates prevalent issues with errors and inadequate training among clinicians, particularly junior doctors. Newly graduated doctors in countries like Sudan are often expected to write most hospital prescriptions and prescribe multiple times per day on hospital drug charts. This study investigated the prescribing competencies of final-year medical students in Gezira State, Sudan, aiming to identify gaps in knowledge, skills, and students’ self- assessment of their preparedness.

**Methods:**

A descriptive cross-sectional study was conducted in 2022. Four medical schools (two governmental, two private) participated, and 162 of 213 eligible students completed the assessment (76% response rate). The validated assessment tool included 55 multiple-choice questions (weighted to 60 points), covering pharmacology, clinical pharmacology, and pharmacotherapy (CPT). Student performance was classified using a criterion-referenced system: Excellent (50–60), Very Good (40–49), Good (30–39), Poor (< 30).

**Results:**

The study revealed a significant deficiency in essential prescribing competencies. Only 17% of students achieved a score of 50% or above in the assessment. The average clinical pharmacology and therapeutics (CPT) knowledge score of students was 22 (± 7.5) out of 60. The students’ performance was lowest in the domains of drug-drug interactions, drug-disease interactions, and identification of prescribing errors with students scoring an average percent 18, 31, and 18%, respectively. Students obtained an average percent score of 36, 40, and 48% regarding drug indications, drug-related side effects, and mechanism of action, respectively. Many students (48%) expressed low confidence in their prescribing skills and dissatisfaction with the amount and quality of undergraduate teaching in (CPT).

**Conclusion:**

Our results suggest that undergraduate teaching of CPT is inadequate in these schools potentially leading to unsafe prescribing. We recommend that CPT curricula should be reviewed and developed under the supervision of qualified clinical pharmacologists to achieve competency-based outcomes. Moreover, we recommend that medical schools should test prescribing competencies of their students prior to graduation.

## Introduction

Prescribing is a demanding and complex process that is becoming increasingly difficult. There is evidence of prescribing errors by a variety of clinicians in a variety of contexts, whether due to mistakes, under- or over-prescribing, and inappropriate or illogical prescribing [1–3]. Prescribing medicines is a fundamental part of medical practice. Therefore, defining and reaching an agreement with stakeholders on essential competencies is a requirement for creating adequate and acceptable curricula and assessments [4]. Prescribing competence requires adequate pharmacological knowledge as a prerequisite for safe prescribing practice [5]. Education and the capacity to adapt to ongoing changes in pharmacotherapy are two key factors that determine the effectiveness of a prescriber in a medical setting [2].

It has recently drawn a lot of notice that medication prescription errors are a substantial public health issue [5, 6]. For example, in England, primary care facilities write over one billion prescriptions each year, and prescriptions written by junior doctors have an error rate of 7–10% [7]. Another study aimed to assess prescribing errors reported by community pharmacy professionals in Gondar Town, Northwest Ethiopia, which revealed that the overall prevalence of prescription errors was 75.1% [8]. Recent studies emphasize a global concern regarding suboptimal prescribing competencies among medical graduates. A study identified considerable variation in prescribing preparedness across Canadian medical schools, calling for standardized national curricula [9]. Additionally, other studies highlighted through scoping and systematic reviews that educational interventions, including e-learning and simulation, can significantly improve students’ confidence and prescribing accuracy [10, 11]. This aligns with broader educational trends advocating for structured, competency-based clinical pharmacology training. A study in Sudan has shown that prescription errors are common and multifactorial [12].

Surveys show medical students, and foundation-year doctors lack confidence in prescription writing, pharmacological knowledge, and shared concerns about inadequate undergraduate education to reduce patient risks [13]. UK medical graduates lack skills in prescribing, emergency management, and error/safety incidents, necessitating educational interventions like interdisciplinary teamwork, prescribing, and clinical reasoning [14]. Physician prescribing decisions are heavily influenced by the pharmaceutical industry, and medical students may lack the necessary knowledge to manage interactions. Identifying educational programs to improve knowledge, attitudes, and skills is crucial, as global regulations vary [15].

Prescribing is one of the most common duties expected of new doctors. This involves a combination of knowledge, judgment, and skills [16]. Preparing them as rational prescribers, a goal set by most medical schools, is of utmost importance, and has proven challenging. In Sudan, the healthcare system has deteriorated noticeably in recent years [17]. Improving it is multifactorial but relies greatly on capacity building as a critical strategy, as shown in many parts of the world [18]. There is a pressing need for pedagogical innovation in clinical pharmacology education in Sudan. Incorporating interactive, case-based, and technology-assisted learning methods of learning were recommended [19] and supported by evidence from study conducted on medical students in clinical pharmacology education at the University of Jeddah [20], could help bridge the gap in competencies. Without such reform, the risk of unsafe prescribing practices among new graduates will likely persist. Hence, assessing whether students attain the desired competencies, including the prescribing skills required for new doctors, is of great importance. There is no formal description of CPT education in medical schools in Sudan. Clinical pharmacologists involved in CPT education in these schools indicate that basic pharmacology is generally taught during the preclinical years using lecture-based approaches, focusing on pharmacokinetics, pharmacodynamics, and mechanisms of action. Clinical pharmacology and pharmacotherapy are introduced later, often during the clinical years, but are commonly delivered through didactic lectures with limited integration into clinical practice. Assessment is primarily knowledge-based using written examinations, with minimal structured evaluation of real-world prescribing skills. Formal revisiting of prescribing competencies during the final year is limited, and standardized prescribing competency assessments prior to graduation are generally lacking. Thus, this study was conducted to assess the prescribing competencies of final year medical students in Gezira State, Sudan.

## Methods

### Study setting and design

Gezira State is in the center of Sudan with a population of more than five million people. There are three governmental and six private schools of medicine in the state. Five medical schools (two governmental and three private) were approached for participation. Four schools agreed to participate, while one school declined after students refused to complete the assessment, citing perceived difficulty of the test. Data collection and analysis were therefore conducted using data from four participating schools only. A descriptive, cross-sectional study was carried out between June and December 2022. Schools were selected based on accessibility, willingness to participate, and availability of final-year students who had completed the internal medicine clerkship and if Institutional permission was granted from the respective administrations.

### Study population

Five medical schools (Faculty of Medicine University of Gezira, Faculty of Medicine University of Al Butana, Medicine and Surgery Program Wad Medani College of Medical Sciences and Technology, Medicine and Surgery Program Al Gezira College for Medical Sciences and Technology and Medicine and surgery program Iqraa College of Sciences and Technology) met the inclusion criteria of having final-year medical students who completed their clerkship in internal medicine. The study was set to assess the prescribing competencies related to four common diseases in Sudan namely hypertension, diabetes mellitus, malaria, and tuberculosis. The final stage of teaching and training of these diseases and their management is conducted during the clerkship of internal medicine across all medical schools in Gezira State. Hence, completion of this clerkship was set as the inclusion criteria.

### Sample size and sampling

The total number of final-year students in the included schools was obtained from the registration offices of each respective school. The names of the schools were coded for confidentiality and anonymity. School 1 had 100 students, school 2 had 80 students, school 3 had 80 students, school 4 had 80 students and school 5 had 115 students. The sample size was calculated using the proportion-based sample size formula with finite population correction. This approach was selected because prescribing competence is inherently threshold-based and because reliable estimates of the population standard deviation for prescribing scores were unavailable. An assumed proportion of 50% was used to provide a conservative and adequately powered estimate. Based on the data collected from the schools’ registrars, the total number of final-year students was 472. According to the included schools, the study population was divided into five strata, and the sample size was determined based on the strata size. The sample size within each stratum was calculated (school 1 (45), school 2 (36), school 3 (45), school 4 (36) and school 5 (51)), and participants were selected via systematic sampling. Weighting was used to ensure that each medical school had the same influence in the descriptive analysis. The initial calculated sample size was 213 students, based on the total eligible population across the approached schools. Of these, 162 students completed the assessment.

### Design of the assessment tool and questionnaire

This tool was designed to assess prescribing competencies that every graduate should have. It was a paper-based assessment tool and questionnaire (in English). It was adapted from the work of Brinkman et al., 2017 [5]. A committee composed of the researcher, an assistant professor in clinical pharmacology and a professor of internal medicine designed the framework for the competency test based on four diseases that were abundantly covered in medical school curricula namely, the chronic diseases diabetes and hypertension and the endemic diseases malaria and pulmonary tuberculosis. The assessment tool consisted of three parts: demographic characteristics, knowledge and skills-based multiple-choice questions assessing prescribing competency across different clinical scenarios, and students’ attitudes and self-confidence regarding prescribing and CPT teaching. The knowledge part consisted of 55 multiple-choice questions (MCQs), some questions weighted to yield a maximum total score of 60, allowing domain-based scoring and expert benchmarking. The MCQs covered drug indications, side effects, mechanisms of action, drug-drug interactions, drug-disease interactions, prescribing errors and contraindications of commonly prescribed drugs. The description of the overall score as (excellent, very good, good and poor) was determined using a criterion referenced system. Excellent (50–60), very good (40–49), good (30–39) and poor (less than 30).

Although prescribing competence ideally includes practical prescribing skills such as prescription writing, dose adjustment, and therapeutic decision-making in clinical contexts, the present assessment primarily evaluated applied prescribing knowledge, including drug indications, identification of contraindications, drug–drug and drug–disease interactions, adverse effects, and prescribing errors. These domains represent essential cognitive components of prescribing competence and are necessary prerequisites for safe prescribing. In addition, self-reported confidence items were included to assess students’ perceived preparedness in performing prescribing tasks such as dose calculation, prescription writing, and monitoring therapy. Due to feasibility constraints and student difficulties encountered during pilot testing, objective structured assessments of practical prescribing skills were not included.

### Validity and reliability of the assessment tool and questionnaire

The face and content validity of the assessment and questionnaire were established during two online modification rounds with the primary investigator. The questions were further assessed by two internal medicine specialists working in academia and clinical practice. The MCQs were administered to a group of 7 clinical pharmacologists and 3 internal medicine specialists working in Wad Madani. The construct validity of the MCQs was based on the scores of clinical pharmacologists and internal medicine specialists. With an average score of 71.6% (SD 3), the internal consistency of the MCQs was good (Guttman Lambda 2 of 0.64). Further modifications to the MCQs were made, and the questionnaire was piloted on 10 final-year medical students from Khartoum State to evaluate clarity, relevance, and feasibility. These students were not included in the final study sample, and pilot results were not included in the analysis.

### Data collection

The data was collected at each school by appointment with the dean/ program coordinator after providing a copy of the research proposal, ethical approval and a letter from the primary investigator explaining the aims of the study and requesting the school’s participation. After approval and written agreement to participate in the study, students were informed about the assessment three days in advance to allow coordination with the medical schools and scheduling of suitable assessment sessions. Students were asked to complete the assessment and questionnaire within 90 min. At the start of the assessment, students were informed about the study objective and received instructions. They were not allowed to use references or mobile phones or to consult each other, or the test invigilator. Participation was voluntary, anonymous, and without consequences. The researcher provided paper-based tests to all students who attended on the arranged date and time.

### Data management and analysis

The Statistical Package for Social Sciences (SPSS) version 26 software (Armonk, NY: IBM Corp.) was used to analyze the data. Data with a p value of 0.05 or less was considered statistically significant. All MCQs were scored as right or wrong (1–0). The pass rate was calculated as the percentage of scores that were more than the half (30).

### Ethical considerations

The study was conducted in agreement with the recommendations of the Declaration of Helsinki and approved by the Ethics Committee of the University of Gezira (No 20–22). Prior to the conduction of the study, written informed consent was obtained from all subjects involved in the study separately, and voluntarily after explaining the purpose of the study in clear and simple words, and confidentiality was maintained throughout the study.

## Results

Data were collected from four medical schools. One school was excluded due to the refusal of participation by its students. Only 162 students completed the assessment, resulting in a response rate of approximately 76% among participating schools. All results presented in this study are based on data from the four participating schools. Most of the students were female (68%). The mean age of students was 23 (SD ± 0.8). All of the participating medical schools were asked to provide information regarding the structure of CPT teaching, teaching methods, contact hours, qualifications of teaching staff, assessment strategies, and perceived preparedness of students for prescribing upon graduation.

### Knowledge and skills of students towards CPT

**Fig. 1:**
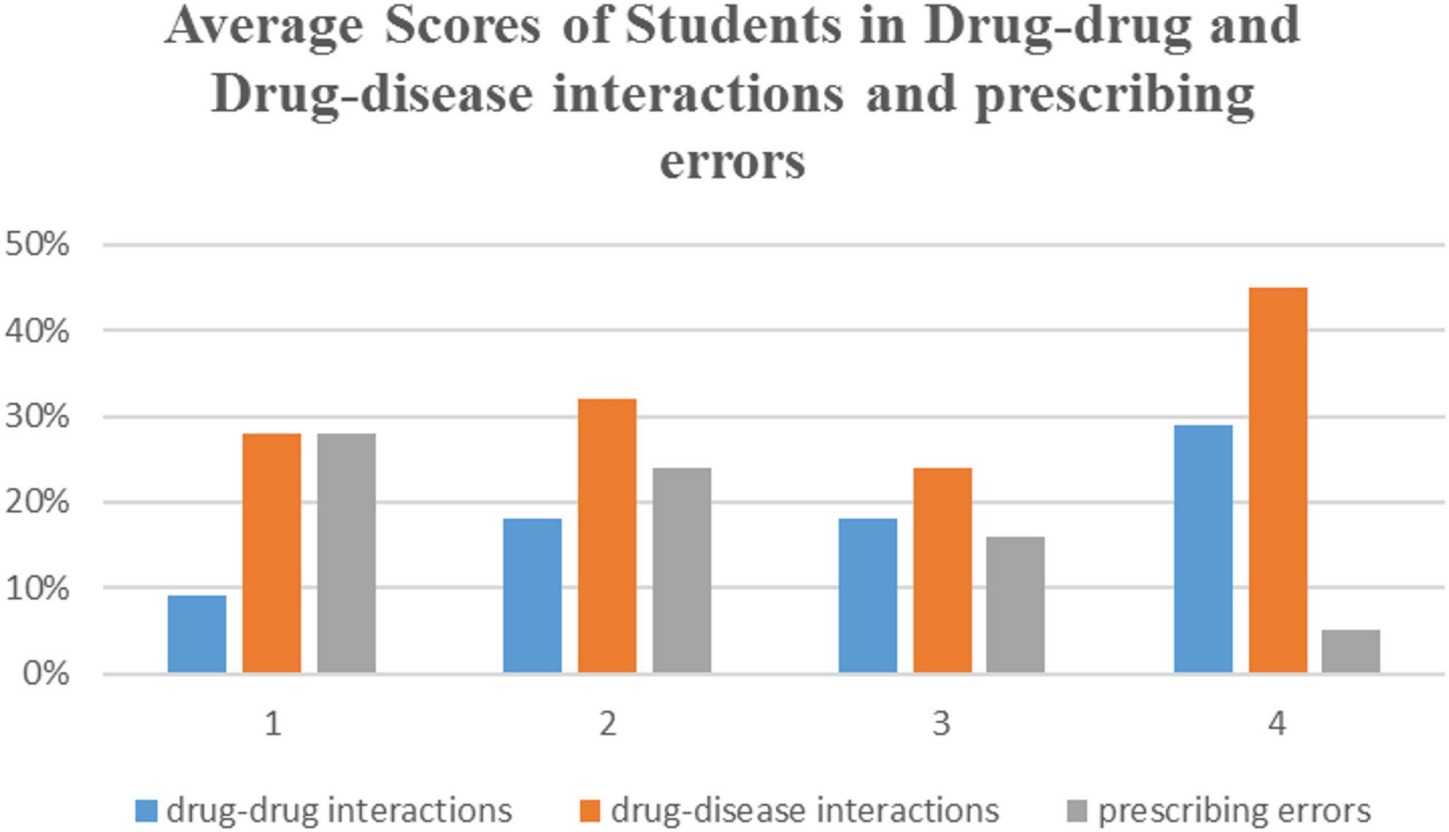
Average scores of students in drug-drug and drug-disease interactions and prescribing errors

The mean overall knowledge score among students was 22 out of 60 (36.6%) (SD ± 7.5), which falls within the “Poor” category according to the predefined criterion-referenced grading system. Only 17% of students achieved the competency pass rate. with an indication score of 36% ± 2, side effects score of 40% ± 2.2, mechanism of action score of 48% ± 2.1, prescribing errors score of 18% ± 1, and contraindications score of 35% ± 0.8. The students’ performance was lowest in the domains of drug-drug interactions, drug-disease interactions and identification of prescribing errors with students scoring an average percent of 18, 31 and 18% respectively (Fig. 1). The average knowledge score of school 1 was 21 (SD ± 5), school 2 was 26 (SD ± 9), school 3 was 23 (SD ± 8) and school 4 was 21 (SD ± 6). The scores of all schools revealed poor knowledge in all tested prescribing competencies except school 2 which was good at some competencies such as identification of drug indications, mechanism of actions and contraindications. In addition, school 3 was good at identification of drug side effects (Table 1). While the overall curricular structure was broadly similar across schools, minor variations were noted in reported course organization, teaching strategies, and assessment formats. Some schools reported limited use of problem-based learning, seminars, or self-directed learning components in CPT teaching.

**Table 1:** Essential knowledge in clinical pharmacology and therapeutics of final-year medical students (*n* = 162)

Knowledge	Percentage for all students (SD)	School 1	School 2	School 3	School 4	*P*- value
Indication	36% (SD ± 2)	15% (SD ± 1.3)	55% (SD ± 2.3)	37% (SD ± 1.5)	39% (SD ± 2.3)	0.005
Side effects	40% (SD ± 2.2)	32% (SD ± 1.7)	47% (SD ± 2.9)	50% (SD ± 1.8)	34% (SD ± 2)	0.001
Mechanism of actions	48% (SD ± 2.1)	38% (SD ± 2.2)	71% (SD ± 2.2)	39% (SD ± 2.2)	47% (SD ± 1.7)	0.001
Drug-drug interactions	18% (SD ± 1)	9% (SD ± 1.3)	18% (SD ± 1.3)	18% (SD ± 1.4)	29% (SD ± 1.4)	0.004
Drug-disease interactions	31% (SD ± 0.8)	28% (SD ± 0.8)	32% (SD ± 0.9)	24% (SD ± 0.9)	45% (SD ± 0.8)	0.001
Prescribing errors	18% (SD ± 1)	28% (SD ± 1.3)	24% (SD ± 1.3)	16% (SD ± 1)	5% (SD ± 1)	0.01
Contraindications	35% (SD ± 0.8)	28% (SD ± 0.7)	63% (SD ± 1)	24% (SD ± 0.8)	24% (SD ± 0.8)	0.009

These findings indicate deficiencies in applied prescribing knowledge domains that are directly relevant to safe prescribing, particularly in recognizing interactions, contraindications, and prescribing errors, which are critical components of prescribing competence.

### Attitudes of students towards CPT

**Fig. 2:**
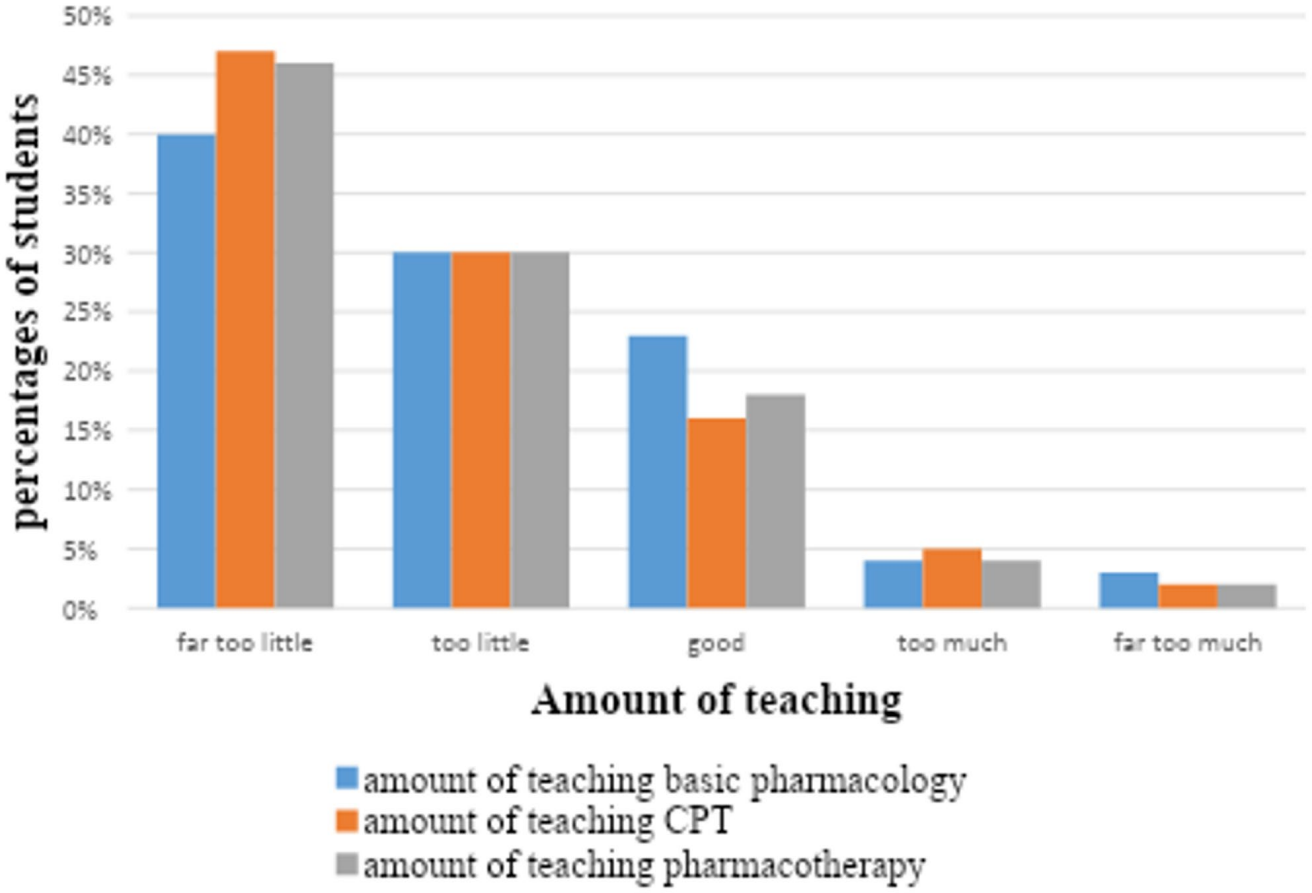
Amount of basic pharmacology, clinical pharmacology, and pharmacotherapy teaching in the undergraduate medical curriculum from the perspective of students

**Fig. 3:**
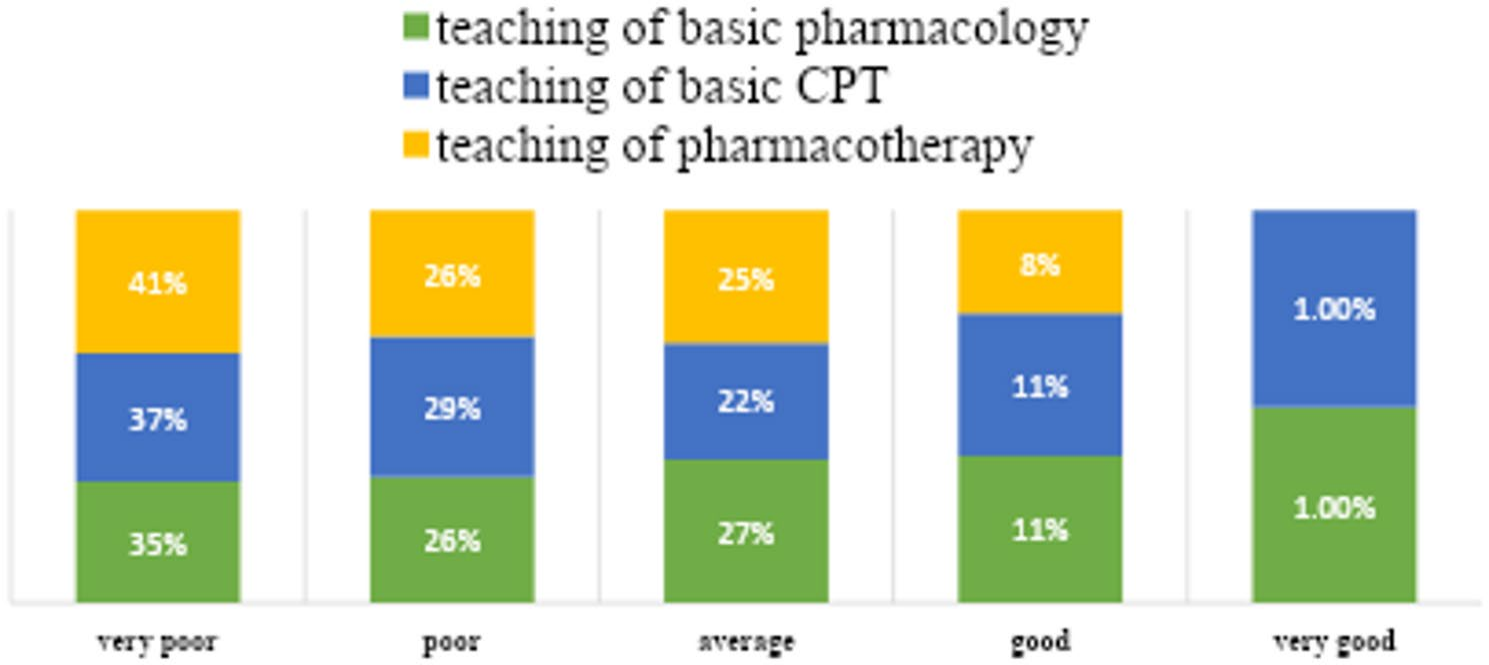
Opinion of students about the teaching process of basic pharmacology, clinical pharmacology, and pharmacotherapy in the undergraduate medical curriculum

Few students felt confident about their prescribing skills (Table 2). Most students (54%) were not satisfied with the amount of undergraduate teaching in CPT they had received and thought that it was too little. And about (7%) of students mentioned that CPT teaching was too much (Fig. 2). Furthermore, more than 50% of students were not satisfied with the undergraduate teaching methods of CPT and thought that the teaching process was poor (Fig. 3). Nearly half of the students (46–48%) reported being very unconfident or not confident in key prescribing skills, including prescription writing and dose calculation. Only 15% felt adequately prepared for their future prescribing responsibilities.

**Table 2:** Self-reported confidence in prescribing skills (*n* = 162)

Prescribing skills	Very unconfident Frequency (%)	Not confident Frequency (%)	Neutral Frequency (%)	Confident Frequency (%)	Very confident Frequency (%)
Taking patient information	15 (9%)	50 (31%)	40 (25%)	40 (25%)	17 (10%)
Specifying therapeutic objective	9 (6%)	47 (29%)	49 (30%)	40 (25%)	17 (10%)
Specifying standard treatment	20 (12%)	40 (25%)	48 (30%)	40 (25%)	14 (8%)
Verifying the suitability of treatment	22 (14%)	53 (32%)	45 (28%)	31 (19%)	11 (7%)
Choosing correct drug	19 (12%)	39 (24%)	46 (28%)	40 (25%)	18 (11%)
Choosing correct dose	20 (12%)	56 (35%)	41 (25%)	36 (22%)	9 (6%)
Calculating dose	30 (19%)	63 (39%)	30 (19%)	21 (13%)	18 (10%)
Writing prescription	21 (13%)	53 (33%)	44 (27%)	31 (19%)	13 (8%)
Giving instructions	24 (15%)	35 (22%)	46 (28%)	41 (25%)	16 (10%)
Determination of monitoring parameters	18 (11%)	48 (30%)	44 (27%)	45 (28%)	7 (4%)

## Discussion

Globally, concerns over the inadequate prescribing competencies of medical graduates have prompted extensive evaluation and curricular reform in many countries. For instance, Brinkman et al. highlighted the need for standardized learning outcomes across Europe to ensure prescriber competencies, and reported widespread deficiencies in clinical pharmacology knowledge, despite significant efforts at the curricular level [1].

In this study, we explored the essential prescribing competencies of final-year medical students in Gezira state, Sudan. Future prescribers should be adequately prepared with prescribing competencies to achieve therapeutic goals. All clinical pharmacologists play a significant role in teaching, whether at the undergraduate, postgraduate, or continuous professional development levels. Undergraduates are currently receiving the most attention due to the rising demands placed on new prescribers, as well as the finding that new prescribers are more likely to prescribe ineffectively and with more errors than their seniors [21].

This study revealed an overall lack of essential prescribing competencies among final-year medical students. In particular, the students had poor knowledge of drug indications, side effects, mechanism of action, drug-drug interactions, drug-disease interactions, prescribing errors, and contraindications according to the scoring system that we used.

Our findings concerning the assessment of prescribing competencies among final-year medical students are an extension of the elegant work by Brinkman et al. [5]. Our results showed that the overall pass rate was 17%. This result revealed an overall lack of knowledge among these students. Our findings showed less knowledge among students than the findings of a study conducted in several European countries, which showed that the overall pass score was higher than the result of our study, but concluded that the overall prescribing knowledge was poor, with an overall knowledge score of 69.6%. As mentioned in that study, the pharmacological mechanism of action knowledge score was 79.3% ± 18.1, side effects score was 79.4% ± 17.7, interactions and contraindications were 50.0% ± 20.9, and prescribing errors score was 54.7% ± 21 [6]. In that study, the total number of students was higher (*n* = 895), and the questions were in all departments of medicine. The substantially lower pass rate observed in this study compared with European studies may be partly explained by differences in teaching quality and quantity. Additionally, while European assessments covered a broad range of medical specialties, the current assessment focused on four common chronic and endemic diseases. Differences in assessment scope may therefore have contributed to the observed variation in performance. In addition, our study results reflected poor knowledge of drug-drug interactions and drug-disease interactions.

Moreover, the knowledge score found in this study was less than that in a study conducted in 31 UK medical schools (*n* = 7343 final-year medical students), where the overall pass rate was 95%. This study was conducted using the Prescribing Safety Assessment tool which was developed as an assessment of competence concerning prescribing and supervising the use of medicines, the questions from all departments of medicine [7]. This assessment was conducted in 2012 and its questions were developed annually. In 2016, all UK medical schools were asked to consider this assessment to their graduates. Before the assessment, the student had access to Prescribing Safety Assessment (PSA) and received general information, 12 information videos, and four 1-h, 30-item practice papers with question-specific feedback. This made the students familiar with the test. Consequently, their knowledge scores were high [7]. The availability of a standard assessment encouraged students to increase their knowledge. In our study, none of the medical schools had a standard prescription assessment. The familiarity of students with such a standard assessment can help them improve their knowledge and obtain better results.

In our study, nearly half of the students felt that their medical curriculum had not adequately prepared them for their future prescribing responsibilities as a junior doctor and the majority of students felt unconfident in the prescribing process, which may be due to their poor academic preparedness. These results were less than those reported in a study of new graduates from Irish medical schools that investigated the level of preparedness for prescribing practice (*n* = 140; female: male 56%:44%). Most respondents felt confident about prescription writing (89%), medication history taking (81%), and accessing drug information in the hospital setting (80%). Only 58% of their respondents felt confident in drug dose calculation and 35% felt confident in preparing and administering drugs. When asked if their undergraduate medical education had prepared them for prescribing in clinical practice, (28%) of respondents agreed and (37%) of respondents agreed that they felt stressed about prescribing medications [22]. These differences in results can be explained by the Irish study, where 82% of medical schools had received formal training in prescribing skills. In our study, the students did not have any formal training in prescribing skills, which may have contributed to their low knowledge and prescribing confidence. However, our results were far better than that mentioned by Brinkman et al., where only 29% of the students felt adequately prepared for their future prescribing tasks as doctors. In addition, more than 25% of the students felt confident in choosing the correct drug, less than 20% in choosing the correct dose, 20% in calculating the correct dose, and writing the prescription was more than 40% [5]. However, their confidence in writing prescriptions was greater than findings of our study.

Students from School 2 demonstrated better performance in certain prescribing domains. This may be related to subtle differences in curriculum organization, greater emphasis on applied pharmacotherapy, or variations in teaching strategies and assessment exposure. However, as this study was descriptive in nature and did not involve a formal curriculum comparison, causal inferences cannot be made.

This study’s findings align with a study in which the investigators observed that fragmented clinical pharmacology teaching contributed to poor retention and confidence among medical students [23]. Furthermore, a scoping review emphasized that educational interventions, such as simulation-based learning and integrated case discussions, significantly improved prescribing confidence and accuracy [10]. These methods are still largely absent in Sudanese medical curricula. Exploring the preference of medical students in clinical pharmacology education underscored the importance of tailoring teaching strategies to student preferences and technological access [20], which may be an effective model for low-resource settings like Sudan.

It is important to note that prescribing competence encompasses both theoretical knowledge and practical prescribing skills. The present study primarily assessed applied prescribing knowledge rather than direct observation of prescribing performance. However, adequate knowledge of drug indications, contraindications, interactions, and adverse effects is a fundamental prerequisite for safe prescribing. Deficiencies in these domains have been consistently associated with increased prescribing errors among junior doctors. Furthermore, students’ low self-reported confidence in key prescribing tasks, such as dose calculation, prescription writing, and treatment selection, supports the conclusion that prescribing preparedness may be inadequate. Nevertheless, future studies incorporating objective assessments of practical prescribing skills, such as clinical simulations or prescription writing exercises, are recommended to provide a more comprehensive evaluation of prescribing competence.

### Study limitations and recommendations

We acknowledge that the competency assessment test did not include a skill part due to inability of students to deal with it in the pilot study. Non-response bias is a further potential limitation. Although 213 students were required, only 162 participated (76%) after one medical school declined involvement due to perceived assessment difficulty, which may have biased competency estimates and limited generalizability. No qualitative component was included to explore in depth why students perceived CPT teaching as insufficient or why they felt inadequately prepared for prescribing. Future studies using qualitative or mixed-methods designs are recommended to better inform curriculum improvement. Also, students’ understanding of the distinctions between basic pharmacology, clinical pharmacology, and pharmacotherapy was not formally assessed prior to administering the questionnaire, which may have influenced their responses. The distinctions were just written in the questionnaire. Another limitation is that the assessment tool primarily evaluated applied pharmacological knowledge and self-reported confidence rather than directly observing practical prescribing performance. While these domains are essential components of prescribing competence, the absence of objective skill-based assessments, such as prescription writing exercises or simulated clinical scenarios, limits the ability to fully evaluate prescribing competence. Future research should incorporate structured assessments of prescribing skills to provide a more comprehensive evaluation. Despite these limitations, the findings of the current study are novel and interesting and highlight the importance of assessing and potentially improving prescribing competencies among medical students. We recommend that more comprehensive studies be conducted to assess prescribing competence and skills at a more national level using more disease models. This should provide evidence of any reforms or steps needed to be taken to ensure a more standardized set of prescribing competences across medical schools. Moreover, we strongly recommend that CPT curriculum should be reviewed and developed with clear learning outcomes under the supervision of clinical pharmacologists.

## Conclusion

To the best of our knowledge, this is the first study in Sudan to assess the prescribing competencies of medical students. There was an overall lack of essential prescribing competencies (drug indications, side effects, mechanism of actions, drug-drug and drug-disease interactions, prescribing errors and contraindications) among final-year medical students. These findings suggest gaps in prescribing preparedness that may increase the risk of prescribing errors if not addressed through improved clinical pharmacology and pharmacotherapy training. Most of the graduates feel unprepared for prescribing and unconfident in dealing with prescribing skills.

## Data Availability

The datasets used and/or analyzed during the current study are available from the corresponding author upon reasonable request.
